# Identification of Gene Associated with Sweetness in Corn (*Zea mays* L.) by Genome-Wide Association Study (GWAS) and Development of a Functional SNP Marker for Predicting Sweet Corn

**DOI:** 10.3390/plants10061239

**Published:** 2021-06-18

**Authors:** Vinitchan Ruanjaichon, Kanogporn Khammona, Burin Thunnom, Khundej Suriharn, Chalong Kerdsri, Wanchana Aesomnuk, Arweewut Yongsuwan, Naraporn Chaomueang, Paradee Thammapichai, Siwaret Arikit, Samart Wanchana, Theerayut Toojinda

**Affiliations:** 1 National Center for Genetic Engineering and Biotechnology (BIOTEC), 113 Thailand Science Park, Pahonyothin Road, Khlong Nueng, Khlong Luang, Pathum Thani 12120, Thailand; vinitchan.rua@biotec.or.th (V.R.); kanogporn.kha@ncr.nstda.or.th (K.K.); burin.thu@biotec.or.th (B.T.); wanchana.a@ku.th (W.A.); golfarweewut@gmail.com (A.Y.); narapornchouw@gmail.com (N.C.); 2 Department of Agronomy, Faculty of Agriculture, Khon Kaen University, Khon Kaen 40002, Thailand; sphala@kku.ac.th; 3 Plant Breeding Research Center for Sustainable Agriculture, Khon Kaen University, Khon Kaen 40002, Thailand; 4 Chai Nat Field Crops Research Center, Chai Nat 17000, Thailand; chalong_maize@live.com; 5 Department of Plant and Soil Sciences, Faculty of Agriculture, Chiang Mai University, Chiang Mai 50200, Thailand; paradee.thammapichai@cmu.ac.th; 6 Department of Agronomy, Faculty of Agriculture at Kamphaeng Saen, Kasetsart University Kamphaeng Saen Campus, Nakhon Pathom 73140, Thailand; siwaret.a@ku.th; 7 Rice Science Center, Kasetsart University Kamphaeng Saen Campus, Nakhon Pathom 73140, Thailand

**Keywords:** maize, kernel sweetness, shrunken2, genome-wide association study (GWAS), single-nucleotide polymorphism (SNP)

## Abstract

Sweetness is an economically important eating quality trait for sweet-corn breeding. To investigate the genetic control of the sweetness trait, we conducted a genome-wide association study (GWAS) in an association panel consisting of 250 sweet corn and waxy corn inbred and recombinant inbred lines (RILs), together with the genotypes obtained from the high-density 600K maize genotyping single-nucleotide polymorphism (SNP) array. GWAS results identified 12 significantly associated SNPs on chromosomes 3, 4, 5, and 7. The most associated SNP, AX_91849634, was found on chromosome 3 with a highly significant *p*-value of ≤1.53 × 10^−14^. The candidate gene identified within the linkage disequilibrium (LD) of this marker was *shrunken2* (Zm00001d044129; *sh2*), which encodes ADP-glucose pyrophosphorylase (AGPase), a 60 kDa subunit enzyme that affects starch metabolism in the maize endosperm. Several SNP markers specific to variants in *sh2* were developed and validated. According to the validation in a set of 81 inbred, RIL, and popular corn varieties, marker Sh2_rs844805326, which was developed on the basis of the SNP at the position 154 of exon 1, was highly efficient in classifying *sh2*-based sweet corn from other types of corn. This functional marker is extremely useful for marker-assisted breeding in *sh2*-sweet corn improvement and marketable seed production.

## 1. Introduction

Sweet corn (*Zea mays* L. *var. rugosa* Bonaf.) is globally an economically important crop, and it is widely used for human consumption from both immature and mature kernels as fresh, canned, and frozen due to its high sugar content [1,2,3]. The demand for sweet corn has been rising on a yearly basis due to enhanced consumption and the increasing availability of food-processing industries. Corn kernels come in different sizes and shapes depending on types and varieties. Sweet-corn kernels are usually more yellow than orange-tinted field corn kernels and are visibly shriveled when dried. The wrinkled appearance in dried kernels is due to a sugary gene that retards the conversion of sugar into starch during endosperm development and results in kernel sugar accumulation [4,5]. 

Sweet corn can be classified on the basis of gene composition in the endosperm into five types: normal, sugary enhanced, super sweet, synergistic, and augmented shrunken [6]. Normal sweet corn contains an allelic mutation in the sugar gene, *sugary1* (*su1*), which increases sugar content, but has a high starch conversion rate. Supersweet corn carries a *shrunken2* (*sh2*) mutant, which maintains high sugar level, has a steady rate of starch accumulation, and is suitable for shipping purposes. *Shrunken2* (*Sh2*) encodes large regulatory subunits of the heterotetrameric ADP-glucose pyrophosphorylase (AGPase) present in the maize-kernel endosperm, which is upstream in the starch biosynthetic pathway [7]. It is a rate-limiting enzyme in starch biosynthesis [8,9]. The mutation at the *Sh2* locus causes corn to be supersweet and has thus been widely used for the development of extrasweet corn cultivars [10]. Maize with the *sh2* mutant has three- and eightfold increases in sugar content (29.9% sucrose) compared with maize with *su1* mutant (10.2% sucrose) and normal corn, respectively [6,11,12,13]. Sugar-enhanced (*se*) sweet corn with an *se* mutant in an *su1* background has a higher sugar level, more tenderness, and an extended shelf life compared with normal sweet corn [14]. Synergistic corn, possessing a gene combination of *su-1*, *sh2*, and *se*, gives a balance quality between high sugar content and tenderness [6,12]. Molecular markers associated with these genes and other sweetness-related genes could help plant breeders to quickly and accurately select the desirable sweetness combination to develop synergistic hybrids. However, functional markers for selecting the sweetness trait in maize are still limited in the public sector and some private companies. Recently, gene-based markers for *shunken2-Reference* allele have been developed and used in marker-assisted sweet corn (*Zea mays Sachharata*) breeding program [15].

Quantitative trait locus (QTL) analysis is extensively used to improve the quality of numerous crops such as rice, wheat, tomato, and soybean [16,17,18,19]. Most QTL analyses are performed by linkage mapping on the basis of a segregating population from a biparental cross. Alternatively, genome-wide association study (GWAS) is a powerful tool to find associations between natural phenotypic diversity and underlying genetic variants [20]. GWAS is a powerful approach to associate natural phenotypic diversity with underlying genetic variants. It has since gained popularity as a valuable tool in the quest for QTLs or candidate genes from selected genetic resources without having to develop mapping populations, which can be labor- and time-intensive. Since the release of the maize B73 reference genome [21], GWAS is routinely used to detect single-nucleotide polymorphisms (SNPs) associated with various developmental and agronomic traits in maize such as plant height [22], tassel architecture [23], major ear [24], and kernel nutritional traits [25,26]. 

In this study, we identify the gene associated with the kernel sweetness trait through a GWAS approach using a collection of 250 sweet and waxy corn inbred and recombination inbred lines (RILs). The set of SNPs, obtained from an Axiom® Maize 600k Genotyping Array, were used to evaluate linkage-disequilibrium (LD) decays, population structure, and SNPs associated with kernel sweetness. Additionally, gene-based SNP markers were developed and validated. SNP markers are extremely useful for maize-breeding programs for sweetness traits.

## 2. Results

### 2.1. Trait Evaluation, Genotyping, and Population Study in Panel of 250 Maize Lines

**Figure 1:**
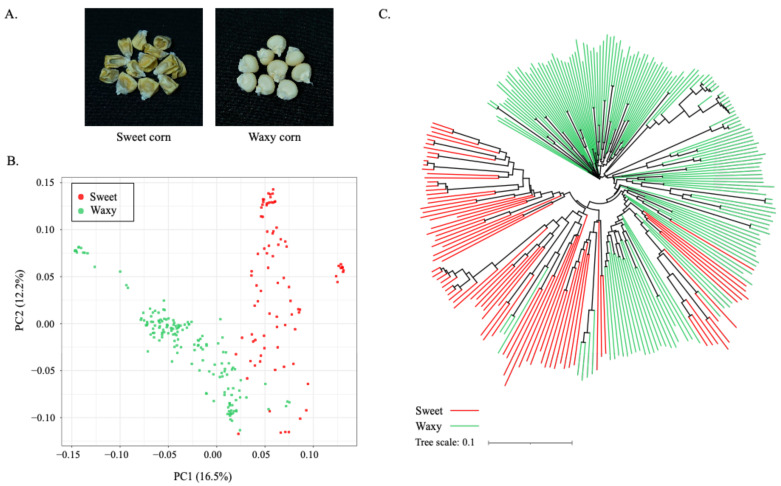
Genetic structure of 250 sweet- and waxy-corn lines. (**A**) Appearance of kernel structures that was used as a criterion to assign maize lines as sweet corn and waxy corn. (**B**) Principal-component analysis plots based on 159,201 SNP markers; (**C**) phylogenetic-tree clustering based on 159,201 SNP markers. The waxy corn and sweet corn accessions in (**B**,**C**) are shown in green and red, respectively.

A total of 250 maize inbred and recombinant inbred lines (RILs) were used to evaluate the kernel trait (Table S1). The phenotype of these maize lines was determined as sweet or waxy corn on the basis of sucrose content and the structure of dried kernels (Figure 1A; Figure S1; Table S1). Among the 250 lines, 86 lines were classified as sweet corn, and 164 lines as waxy corn (Table S1). The sucrose content in the sweet corn group varied from 23.33 to 88.71 mg/g fresh weight with an average of 43.61 mg/g, while the content in waxy-corn group varied from 3.46 to 15.59 fresh weight with an average of 7.70 mg/g (Figure S2). We then genotyped these 250 maize lines using the Axiom^®^ Maize 600 K Genotyping Array (616,201 variants) to obtain SNP data for the population study and GWAS analyses. Out of 616,201 variants, 426,858 high-quality SNPs were determined in the 250 lines. All of these SNPs had a minor-allele frequency (MAF) greater than 0.05, and maximal missing rate of less than 20%. These numbers of SNPs were further filtered to obtain a manageable SNP dataset. As a result, a subset of 159,201 SNPs were retained after filtering on the basis of the distance of at least 1 kb between each pair of adjacent SNPs (Figure S3); 19,565 SNPs were retained after linkage-disequilibrium (LD) pruning. Determined on the basis of 159,201 SNPs, heterozygosity among maize lines varied from 0.02 to 0.20, with an average of 0.09 (Table S1). 

We performed principal-component analysis (PCA) on the basis of the 159,201 SNPs to characterize the population structure, and performed phylogenetic-tree analysis to determine the genetic diversity of the 250 maize lines. PCA revealed clearly separated sweet and waxy corn along the first principal component (PC1; Figure 1B). The phylogenetic tree also identified two major clusters corresponding to waxy and sweet corn, similar to those revealed by PCA (Figure 1C). In addition, on the basis of the 19,565 LD-pruned SNPs, STRUCTURE identified four subpopulations among the 250 maize lines (Figure S4). 

**Figure 2:**
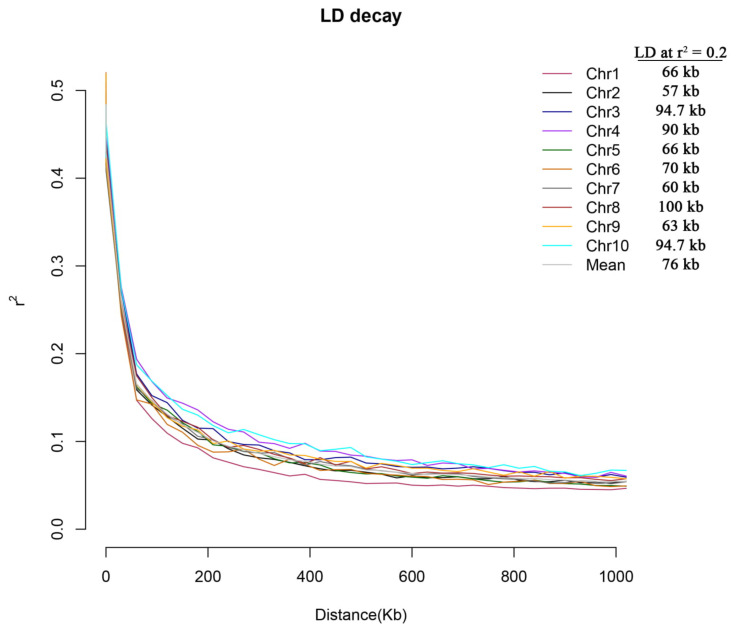
Overall chromosome-wide linkage-disequilibrium (LD) decay estimated from single-nucleotide polymorphism (SNP) genotypes of 250 maize lines. Each line plot represents a smoothed R^2^ for all marker pairs on each chromosome depending on the distance between marker pairs.

We further analyzed the genome-wide LD decays among the 250 maize lines using the 159,201 SNPs. Results showed that the overall LD decay across the genome of 250 maize lines was 210 kb at a cut-off r^2^ = 0.1; the mean length of LD decay decreased rapidly to 76 kb at a cut-off of r^2^ = 0.2 (Figure 2).

### 2.2. Genome-Wide Association Study (GWAS) for Sweetness Trait

**Figure 3:**
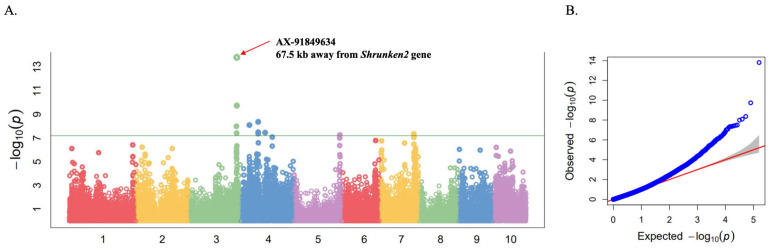
GWAS results for sweetness trait in 250 sweet- and waxy-corn inbred and recombinant inbred lines (RILs), genotyped with 159,201 SNPs, using a mixed linear model implemented in GAPIT. (**A**) Manhattan plots. Each dot represents a SNP. Bonferroni threshold of –log_10_ *p*-value = 13.81 is presented by a green line on Manhattan plots. Most associated SNP AX_91849634, located 67.5 kb on chromosome 3 near the *Shrunken2* gene, is indicated by a red arrow. (**B**) Quantile–quantile (Q–Q) plots. The plot shows the expected vs. observed –log_10_(*p*) of each marker (blue dots). Red line is a guide for the perfect fit to expected –log_10_(*p*). The gray shaded area shows the 95% confidence interval for the Q-Q plot under the null hypothesis of no association between the SNP and the trait.

We performed a GWAS for the sweetness trait in a panel of 250 inbred and recombinant inbred lines (RILs) on the basis of the 159,201 SNPs using a mixed linear model (MLM) implemented in the Genome Association and Prediction Integrated Tool (GAPIT version 3.0). SNP-based heritability for sweet corn versus waxy corn was 99.97%. The GWAS result identified 12 significant SNPs located on chromosomes 3, 4, 5, and 7 that passed the Bonferroni threshold of –log_10_ *p*-value = 7.20 (Figure 3; Table 1). The most associated SNP (AX_91848634) with a –log_10_ *p*-value of 13.81 was located on chromosome 3. Three more significant SNPs located on this chromosome were AX_90850969, AX_91593662, and AX_91593878, which were also detected in the region close to AX_91848634 (Table 1). These four significant SNPs had minor-allele frequency (MAF) varying in the range of 0.31–0.33. The eight other significant SNPs—five of which located on chromosome 4, one of which located on chromosome 5, and two of which located on chromosome 7—mostly had a low MAF value that varied in the range of 0.05–0.09, except for AX_90866628, located on chromosome 4, which had an MAF of 0.48 (Table 1). The defined approximately 100 kb interval of the most associated region on chromosome 3 contained *Shrunken2* (*Sh2*), a gene involved in kernel starch biosynthesis (Table 1). The lead SNP (AX_91848634) was 67.5 kb away from *Sh2*. The gene was located within the LD (210 kb; r^2^ = 0.10) of the lead SNP. No starch- or sugar-related genes were annotated within the significant regions on the other detected chromosomes (Table S3). Based on these results, *sh2*, which is known to control sweetness in super-sweet corn, was considered as the candidate gene associated with the sweetness trait in this maize panel.

### 2.3. Development of SNP Markers Associated with Sweetness Trait Based on Sh2 Variants and Validation among Different Maize Types 

**Figure 4:**
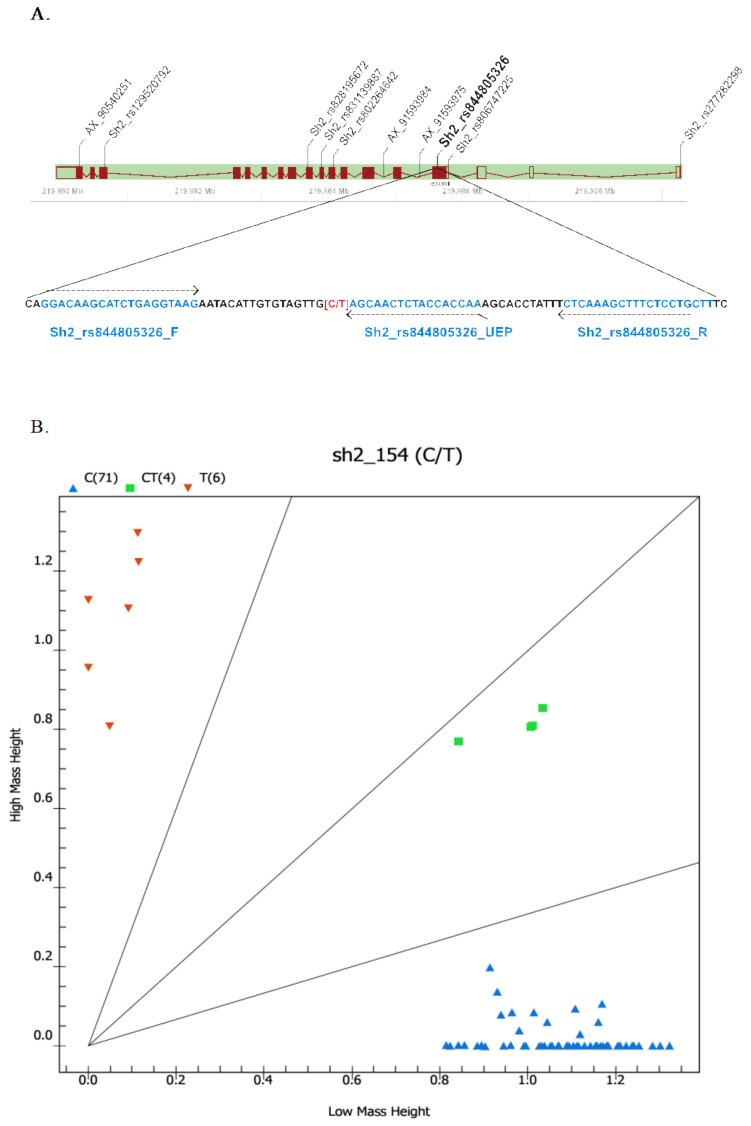
MassArray marker associated with sweetness trait developed based on the SNP in *sh2*. (**A**) Physical locations of molecular markers developed on the basis of gene *Sh2*. Exons and introns in the gene structure are presented by filled boxes and lines, respectively. Unfilled boxes represent untranslated regions (UTRs). Primers (dashed arrows) are presented along with the target sequence. The letters F and R in the primer’s name indicate forward and reverse primers, respectively. UEP indicated an unextended primer used in a single-base primer extension reaction. (**B**) Allelic discrimination plot of marker Sh2_rs844805326 (SNP 154_CT) validated in panel of 81 lines consisting of different types of corn analyzed by MassARRAY^®^ platform. Scatter dots with different colors show clustering of homozygous genotype TT (brown), heterozygous genotype CT (green), and homozygous genotype CC (blue).

To verify the association between *Sh2* and the sweetness trait in this maize panel, we developed SNP markers on the basis of variants in the *Sh2* gene and genotyped in a subset of the maize lines used in GWAS analysis. Ten of the SNP variants on the gene were selected to develop the SNP markers; seven variants were retrieved from the Gramene (https://ensembl.gramene.org, accessed on 1 May 2021) (gene id: Zm00001d044129), and three variants were obtained from the Affymetrix Axiom 600 K genotyping array (Table 2; Figure 4A). Among these, six SNPs had a missense effect, two SNPs were located on the introns, and two SNPs were located upstream the first start codon (Figure 4). These SNPs are different from those used to develop the gene-based markers for *sh2-Reference* allele [15]. The markers were designed on the basis of these 10 selected SNPs and validated in a set of 43 maize lines using a MassARRAY^®^ platform (Table S4). According to regression analysis for marker-trait association for these 10 markers, marker Sh2_rs844805326 exhibited the most association with the trait (R^2^ value of 1.00, *p* < 0.000) (Table S5). This marker was developed on the basis of the SNP variant (C/T) located at exon 1 at position 154 of the coding sequence (CDS) (Figure 4A). Among the 43 maize lines, 38 lines with the sweet-corn phenotype contained an SNP (C), and five lines with the waxy-corn phenotype contained an SNP (T). The SNP variant at this position caused an amino acid change from threonine to alanine at position 52 of the amino acid sequence.

The SNP marker was then used to genotype a set of 81 diverse maize composing of hybrids, inbred lines, and RILs (Table S2). As expected, this marker clearly separated the *sh2*-based sweet corn from waxy corn, field corn, and the sweet corn that was based on the other genes, i.e., *su1, se1,* and *bt2* (Figure 4B). The sweet-corn hybrids (*sh2*/*se*) exhibited a heterozygous genotype using this marker.

## 3. Discussion

Sweet corn is a globally popular crop for human food and the seed-production industry. Sweetness in corn kernels is controlled by six recessive mutant genes expressed in the endosperm: *brittle1* (*bt1*), *brittle2* (*bt2*), *shrunken1* (*sh1*), *shrunken2* (*sh2*), *sugary1* (*su1*), and *sugary enhancer1* (*se1*) [1,2,3,4,5]. One gene in particular, *shrunken2* (*sh2*), which causes a significant increase in endosperm sugar level, is extensively used in sweet-corn cultivar development. In this study, we performed GWAS analysis against the sweetness and waxy characteristics in a set of maize inbred and recombinant inbred lines. The used population in this study clearly showed distinct kernel phenotypes as sweet corn with a shriveled kernel, and waxy corn with an opaque kernel. Therefore, the two characteristics were simply determined as a qualitative trait using a binary code of 0 and 1 for sweetness and waxy, respectively, for GWAS analysis. Although GWAS is usually aimed at quantitative traits, GWAS based on qualitative traits were also reported [27]. Individuals in the sweet-corn group could contain a mutant in *sh2*, *se1, su1,* and *bt2,* and individuals in the waxy-corn group possibly contained a mutation in the *waxy* gene. However, the exact mutation in these individuals was not clearly characterized. 

Due to the strong population structure and a degree of individual relatedness present in our association panel, we performed association tests on the basis of the mixed linear model (MLM) that incorporated the PCA and kinship matrices. The GWAS results accordingly revealed that SNP AX_91849634 was significantly associated with kernel type and was located 67.5 kb away from *Sh2* on chromosome 3. However, no significant SNP was detected for other genes related to the maize endosperm, such as *brittle2* (*bt2*), *sugary1 (su1*), *sugary enhancer1* (*se1*), and *waxy* (*wx*). The lack of a significant association might be compressed by the amount of sweet-corn type in the genetic resource panel that is commonly controlled by the *sh2* gene. This could be due to the fact that the maize lines were preferentially selected and maintained for sweetness as well as other agronomic traits, as these maize lines are used for genetic mapping and breeding purposes. Therefore, it is possible that the lines with *sh2* background were more desirable and hence preferentially selected. 

*Shrunken2* (*sh2*) is extensively used in sweet-corn cultivar development [13]. The *Sh2* gene was previously reported to be involved in starch biosynthesis in the maize endosperm, which encodes the large subunit of the heterotetrameric enzyme, adenosine diphosphate glucose pyrophosphorylases (AGP), and a rate-limiting enzyme [28,29]. A naturally occurring allele, *sh2*, is a loss-of-function mutation that causes a significant reduction in AGP activity [29]. Thus, sucrose could not be converted into ADP glucose, a precursor in starch biosynthesis pathway, resulting in low levels of starch and endosperm sugar accumulation in sweet corn [30]. 

Molecular markers are used for the characterization of germplasms, identification and mapping of genes and QTLs for various agriculturally important traits in maize [31,32,33,34,35,36]. A previous study identified four simple sequence repeat (SSR) markers linked to the *sh2* gene via bulk-segregant analysis (BSA) in three mapping populations [37]. Ideally, markers located on the gene are useful for improving such traits via marker-assisted breeding. However, there is no report of functional markers associated with genes or QTLs underlying kernel sweetness in maize. Chhabra et al. (2020) recently reported gene-based PCR markers that resided in the 5’UTR and intron-12 regions of the *sh2* gene [15]. In this study, we extended the search for gene-based SNPs around our most associated SNP near the *sh2* gene on chromosome 3. One SNP was validated to differentiate sweet corn from other corn types by possessing a *sh2* recessive allele (T). This SNP was located within the coding region in exon 1 at position 154 of the coding sequence, and represents protein sequence conversion from threonine to alanine at position 52 of the amino acid sequence. This might render an enzyme defective due to a change in polarity from a nonpolar, hydrophobic to a polar, uncharged amino acid, thereby affecting starch synthesis in the maize endosperm. The identified SNPs on the *Sh2* gene could be used as selection markers for marker-assisted selection in maize-breeding programs. Moreover, the functional SNP identified on *Sh2* would offer essential information for marker-based breeding in sweet-corn production and seed industry. The gene-based SNP markers identified thus far could effectively save breeders cost and valuable time from lengthy field trials, and aid in accelerating sweet-corn breeding programs.

## 4. Materials and Methods

### 4.1. Plant Materials and Kernel-Trait Evaluation

The association panel consisted of 250 sweet- and waxy-corn inbred and recombinant inbred lines (RILs) originally developed to contain different kernel-trait genes and gene combinations, such as, *su*, *se*, *sh2*, *bt*, *wx*, *btwx*, and *btsh2wx*. Out of 250 lines, 60 were developed by Chai Nat Field Crops Research Center (CNFCRC) and 190 were developed by the Plant Breeding Research Center for Sustainable Agriculture, Faculty of Agriculture, Khon Kaen University (KKU; Table S1). The genotyping panel for marker validation consisted of 81 sweet- and waxy-corn inbred and hybrid lines, including field corn (Table S2). Sucrose content was analyzed from immature ears of each line at 24 days after pollination using the method described previously [38]. Based on the sucrose content together with the structure of dried kernels, the kernel phenotype was defined as a simple qualitative trait and the coding numbers 0 as sweet corn (shrunken kernels with high sucrose content) and 1 as waxy corn (non-shrunken and opaque kernels with low sucrose content) were used for phenotyping.

### 4.2. SNP Array Genotyping and Data Filtering

DNA from the 250 maize lines was extracted from young leaves using DNeasy Plant Mini Kit (QIAGEN) and then genotyped by the Axiom^®^ Maize 600k Genotyping Array that contained 616,201 variants (www.affymetrix.com, accessed on 1 May 2021). SNPs with more than 10% missing data and a minor-allele frequency (MAF) less than 0.05 were removed, yielding a total of 426,858 quality SNPs. Resulting SNPs were then further pruned to retain SNPs of at least 1 kb apart between each pair of adjacent SNPs. Lastly, a total of 159,201 remaining SNPs were retained. These SNPs were used for the genome-wide association study (GWAS) for the kernel trait. Another set of pruned SNPs was performed on the basis of linkage disequilibrium (LD) to obtain a subset of unlinked SNPs using a variant pruning tool (-indep-pairwise 50 10 0.1) in PLINK [39]. A total of 19,565 SNPs were retained. This set of SNPs was used for population-structure analysis using STRUCTURE [40]. The genome coordinates of the SNP loci throughout the study were based on B73_RefGen_V4 (maizegdb.org). 

### 4.3. Population-Structure Analysis and Linkage-Disequilibrium (LD) Decay

Principal-component analysis (PCA) was performed to determine the genetic population structure of the samples using TASSEL 5.0 software [41]. The linkage disequilibrium (LD) between SNPs on each chromosome was estimated with r^2^ using the same software. Genetic distance was calculated using Nie’s standard distance [42], and the phylogenetic tree was created using the CLC Genomics Workbench (QIAGEN, Germany) on the basis of the unweighted pair group with the arithmetic mean (UPGMA) method with 1000 bootstrap replicates. LD-based SNP pruning was also performed to collect representative SNPs that were used for STRUCTURE analysis. STRUCTURE analysis was performed using a Bayesian model-based clustering algorithm implemented in STRUCTURE version 2.3.4 [40], where the admixture model with correlated allele frequencies was used. A total of 10 independent replicates were run for each genetic cluster (K) value (K = 1–10), using a burn-in period of 10,000 and a run length of 10,000 iterations. LnP(D) values were derived for each K and plotted to find the plateau of the ΔK. The final population structure was calculated using the structure harvester [43]. Genome-wide LD decay versus genetic distance was estimated by pairwise analysis of adjacent SNPs within a chromosome using PopLDdecay [44] based on the set of 159,201 SNP markers. 

### 4.4. Genome-Wide Association Study (GWAS)

The GWAS was performed for a simple qualitative trait of kernel structure between sweet versus waxy corn with a mixed linear model (MLM) that incorporated PC and kinship data using R package Genome Association and Prediction Integrated Tool (GAPIT) version 3 [45]. The significant threshold for SNP–trait associations was the Bonferroni correction of *p* ≤ 0.01/n, where n represents the number of SNP markers across the entire genome.

### 4.5. Candidate-Gene Analysis

The genomic regions within the LD block of the significant SNPs (peak SNPs) were selected to identify candidate genes. Gene annotation was based on the B73 reference genome (B73_RefGen_v4). SNP probe sequences of ~150 bp on the Axiom^®^ Maize 600 k Genotyping Array were used as queries in a BLAST algorithm-based search against the reference genome sequence in MaizeGDB (http://www.maizegdb.org/gbrowse; accessed on 1 May 2021) to identify the positions in the maize genome. 

### 4.6. Gene-Based SNP Marker Development and Validation 

Genomic sequences for primer design were obtained from B73 sequence version 4 at Gramene (https://ensembl.gramene.org; accessed on 1 May 2021). The primer set for high-throughput MassARRAY was designed and analyzed according to the manufacture of the MassARRAY^®^ iPLEX system protocol. The primers for the SNP sites were designed to contain 1 bp mismatch at the second base closest to the 3’ end of the forward primer. All markers were validated in the 81 maize accessions, including popular commercial maize varieties (Table S2).

## Figures and Tables

**Table 1:** List of significantly associated SNPs identified by GWAS showing SNP IDs, chromosomes, positions, –log_10_ (*p*) values, minor-allele-frequency (MAF) values, marker R^2^ values, and candidate gene.

SNP	Chr	Position (V4)	–log_10_(*p)* Value	MAF	R^2^	Candidate Gene
AX-91593662	3	218,476,619	7.99	0.33	0.17	-
AX-91593878	3	219,463,930	7.42	0.31	0.16	-
AX-91849634	3	22,005,7126	13.81	0.33	0.18	Zm00001d044129 (*Shrunken2*)
AX-90850969	3	220,099,694	9.74	0.34	0.17	-
AX-90866628	4	42,950,506	8.1	0.48	0.16	-
AX-90877243	4	83,464,190	7.5	0.05	0.11	-
AX-91612113	4	83,465,341	8.37	0.05	0.16	-
AX-91346854	4	83,501,441	7.37	0.07	0.11	-
AX-91617781	4	115,510,176	7.46	0.05	0.17	-
AX-90975122	5	213,164,020	7.26	0.07	0.10	-
AX-91411958	7	159,043,703	7.34	0.09	0.09	-
AX-91740905	7	159,053,605	7.34	0.09	0.11	-

**Table 2:** Details of SNP markers for *Sh2* gene used for genotyping by MassARRAY^®^ platform.

Markers	Chr	Position (V.4)	SNPs	Unextended Primer (UEP) Sequence (5′---3′) ^a^	UEP Mass (Da)	Call 1	Mass 1 (Da)	Call 2	Mass 2 (Da)
AX_90540251	3	219,980,272	G/A	tGTGATCTTGAAGAATGCAA	6180	G	6427.2	A	6507.1
Sh2_rs129520792	3	219,980,689	A/G	ccccgGCTTCTTCTTCAGTTTCATAG	7839.1	A	8110.3	G	8126.3
Sh2_rs828195672	3	219,983,718	C/G	AATTCTTTGAAAAACCAAAGG	6446.2	G	6693.4	C	6733.5
Sh2_rs831139887	3	219,983,940	C/T	gtatcGTGATATCAGCATCGTCCT	7318.8	C	7565.9	T	7645.9
Sh2_rs802264642	3	219,984,110	C/T	CCTGTATTTCTTTAGGATTATTACA	7612	T	7883.2	C	7899.2
AX_91593984	3	219,984,862	T/A	cccgACCACTTCGATTATGG	6052.9	A	6324.2	T	6380
AX_91593975	3	219,985,438	A/T	cccTCAAATAGGGTCATCT	5747.8	T	6019	A	6074.9
Sh2_rs844805326	3	219,985,637	C/T	gTTGGTGGTAGAGTTGCT	5616.6	T	5887.9	C	5903.9
Sh2_rs806747225	3	219,985,795	A/G	AGTGCAAACTGCATATCT	5482.6	C	5729.8	T	5809.7
Sh2_rs277282298	3	219,989,285	G/A	ACCACTACCACCAACA	4748.1	C	4995.3	T	5075.2

^a^ The small letters are the bases added to the 5′ end to adjust the mass of UEPs.

## Data Availability

Data are contained within the article and the Supplementary Materials.

## Supplementary Materials

The following are available online at https://www.mdpi.com/article/10.3390/plants10061239/s1, Figure S1: the physical appearance of dried kernels of sweet, waxy and field corns; Figure S2: box plots and statistical test of sucrose content compared between sweet corn and waxy corn groups in the panel of 250 lines; Figure S3: density and distribution of SNPs throughout 12 rice chromosomes; Figure S4: subpopulations of 250 maize lines; Table S1: list of 250 inbred and recombinant inbred lines used for GWAS analysis in this study; Table S2: list of 81 inbred and recombinant inbred lines, and commercial hybrids used for marker validation; Table S3: annotation of genes located within the same LD block as the associated SNPs. Table S4: list of maize samples used to validate MassARRAY markers. Information on types and sources of maize samples, and genotypes of each marker are provided; Table S5: list of MassARRAY markers designed on the basis of 10 variants in Sh2. Marker details and R2 values from single marker analysis validated on 43 maize lines are provided.
