# Supplementary material for: Identification of Gene Associated with Sweetness in Corn (Zea mays L.) by Genome-Wide Association Study (GWAS) and Development of a Functional SNP Marker for Predicting Sweet Corn
Source: Plants (Basel). 2021 Jun 18;10(6):1239. doi: 10.3390/plants10061239 (PMC8235792; doi:10.3390/plants10061239)
Supplement: Supplementary file 1 [file plants-10-01239-s001.zip › Figure S1 - Corn Kenel structure .pdf]

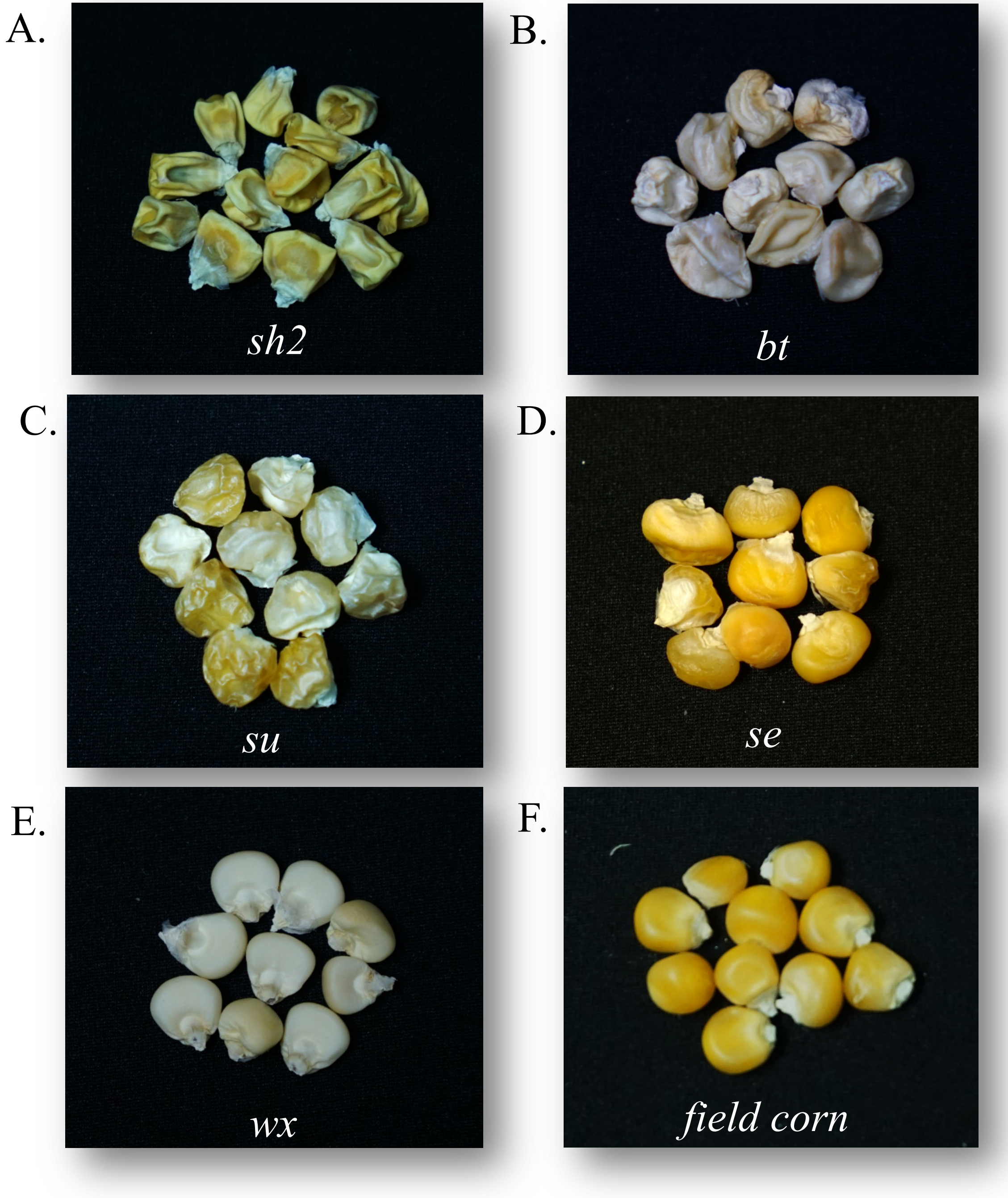

Figure S1. The physical appearance of dried kernels of sweet, waxy and field corns. (A)-(D) represent sweet corn with the background *sh2*, *bt*, *su* and *se*, respectively. (E) and (F) represent waxy corn and field corn (common corn), respectively.
