# Supplementary material for: Identification of Gene Associated with Sweetness in Corn (Zea mays L.) by Genome-Wide Association Study (GWAS) and Development of a Functional SNP Marker for Predicting Sweet Corn
Source: Plants (Basel). 2021 Jun 18;10(6):1239. doi: 10.3390/plants10061239 (PMC8235792; doi:10.3390/plants10061239)
Supplement: Supplementary file 1 [file plants-10-01239-s001.zip › Figure S2 - Boxplots and stats of sucrose content.pdf]

Sucrose content (mg/g fresh weight)

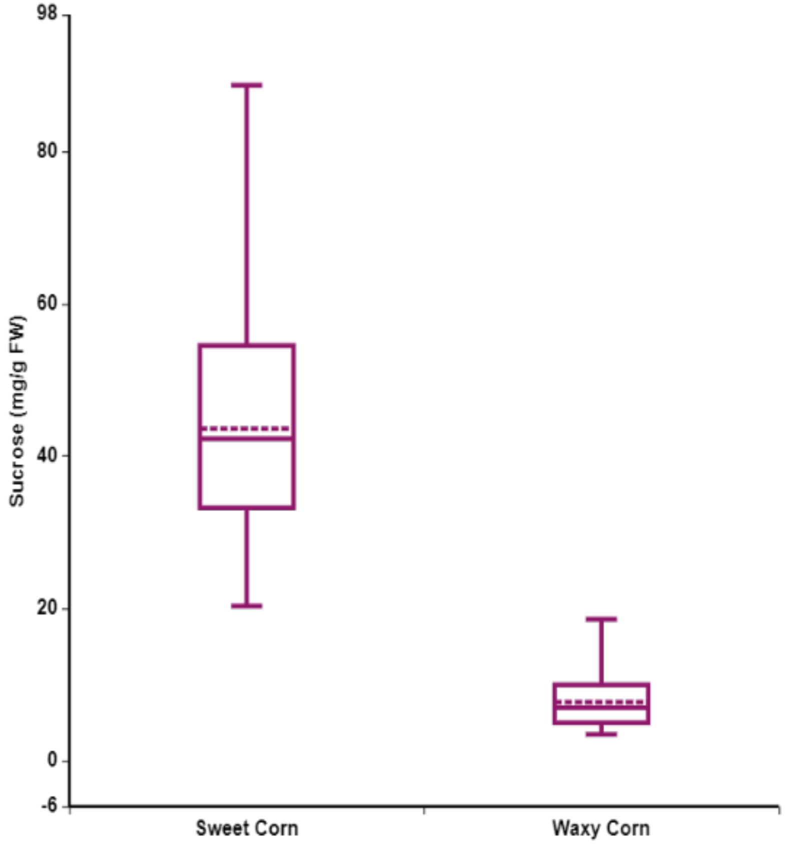

Sucrose content (mg/g fresh weight)

	Sweet corn (n=86)	Waxy corn (n=164)
min	20.334	3.468
max	88.719	18.595
mean	43.617[a]	7.701[b]
% CV	43.235	
LSD (0.05)	24.122	

Figure S2. Boxplots and statistical test of sucrose content comparing sweet corn and waxy corn groups in panel of 250 lines. Comparison of means was conducted with least significant difference (LSD) test ($p < 0.05$) using Agricolae R package.
