# Supplementary material for: Identification of Gene Associated with Sweetness in Corn (Zea mays L.) by Genome-Wide Association Study (GWAS) and Development of a Functional SNP Marker for Predicting Sweet Corn
Source: Plants (Basel). 2021 Jun 18;10(6):1239. doi: 10.3390/plants10061239 (PMC8235792; doi:10.3390/plants10061239)
Supplement: Supplementary file 1 [file plants-10-01239-s001.zip › Figure S4 - STRUCTURE.pdf]

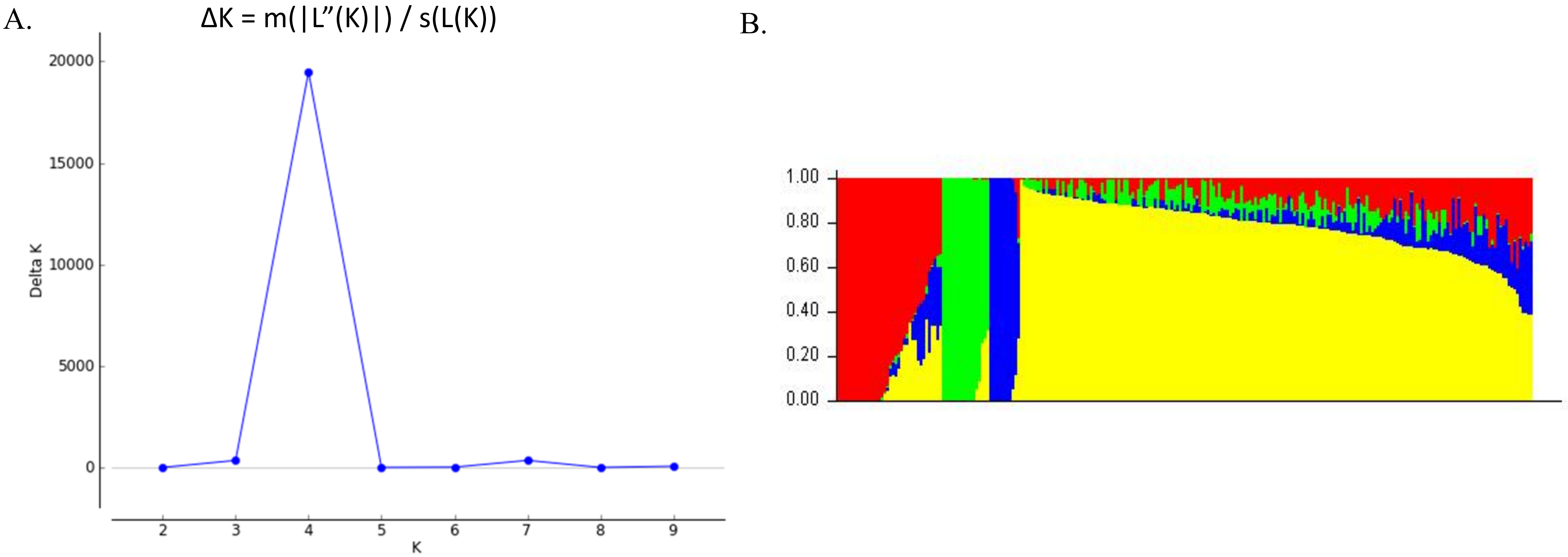

Figure S4. The subpopulations of the 250 maize lines. A. Best delta K estimation by Evanno method; Delta K (ΔK) = m(|L”(K)|)/s[L(K)], where m = means of the absolute values of L”(K), divided by the standard deviation of L (K). B. Estimated population structure of 250 tropical adapted maize recombinant inbred lines as revealed by 19,565 SNP markers for K = 4.
