# Supplementary figures and images for: Identification of Gene Associated with Sweetness in Corn (Zea mays L.) by Genome-Wide Association Study (GWAS) and Development of a Functional SNP Marker for Predicting Sweet Corn
Source: Plants (Basel). 2021 Jun 18;10(6):1239. doi: 10.3390/plants10061239 (PMC8235792; doi:10.3390/plants10061239)

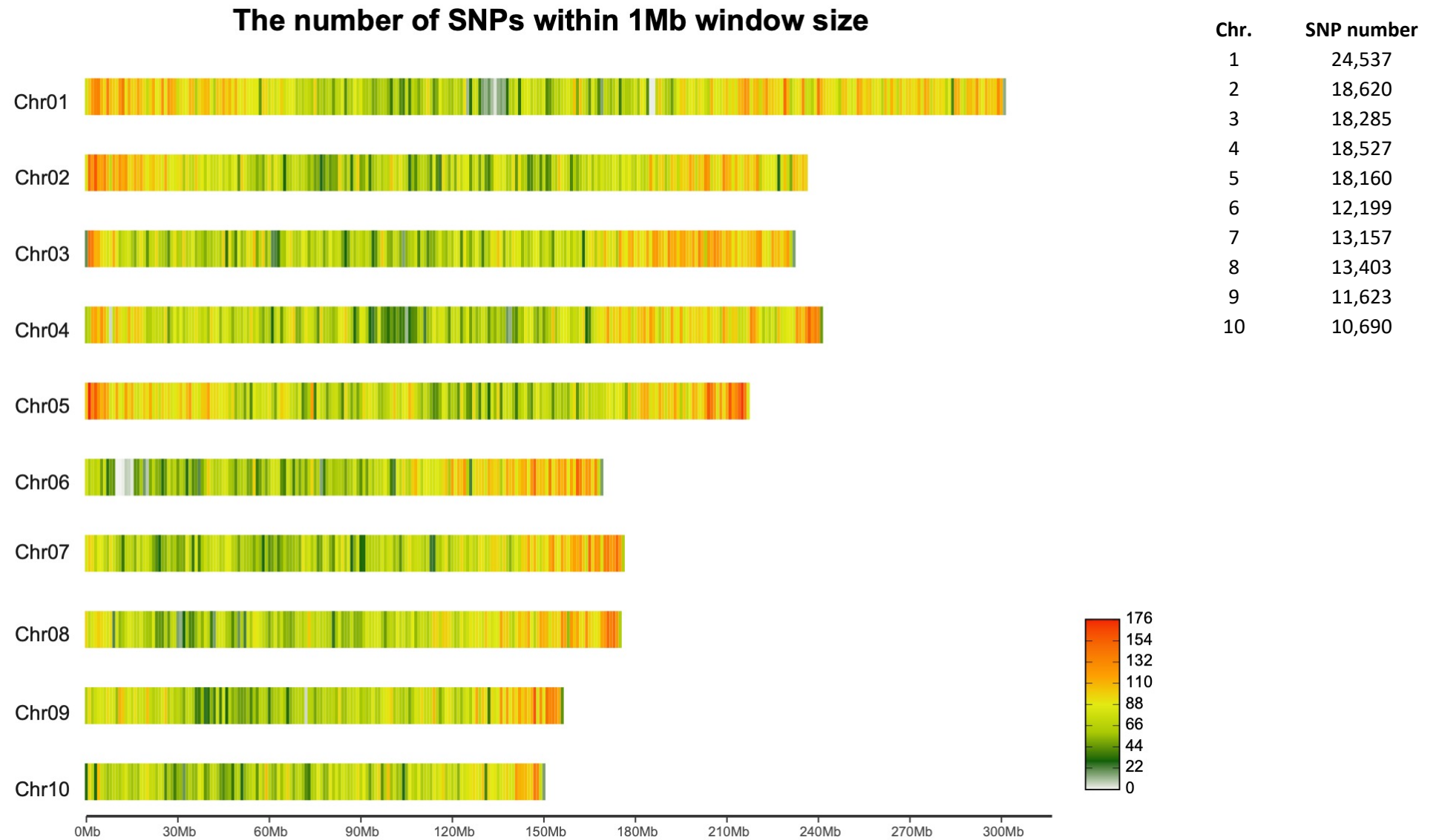

**Figure S3.** Density and distribution of SNPs throughout 10 maize chromosomes

Supplement: Supplementary file 1 [file plants-10-01239-s001.zip › Figure S3 - SNP density.pdf]
